# Weaning from tracheostomy in subjects undergoing pulmonary rehabilitation

**DOI:** 10.1186/s40248-015-0032-1

**Published:** 2015-11-27

**Authors:** Franco Pasqua, Ilaria Nardi, Alessia Provenzano, Alessia Mari, on behalf of the Lazio Regional Section, Italian Association of Hospital Pulmonologists (AIPO)

**Affiliations:** Pulmonary Medicine and Rehabilitation, Villa Delle Querce Hospital, Nemi, Rome Italy; Pulmonary Rehabilitation, San Raffaele Hospital, Montecompatri, Rome Italy

**Keywords:** Mechanical ventilation, Predictive models, Pulmonary rehabilitation, Tracheostomy, Weaning

## Abstract

**Background:**

Weaning from tracheostomy has implications in management, quality of life, and costs of ventilated patients. Furthermore, endotracheal cannula removing needs further studies. Aim of this study was the validation of a protocol for weaning from tracheostomy and evaluation of predictor factors of decannulation.

**Methods:**

Medical records of 48 patients were retrospectively evaluated. Patients were decannulated in agreement with a decannulation protocol based on the evaluation of clinical stability, expiratory muscle strength, presence of tracheal stenosis/granulomas, deglutition function, partial pressure of CO_2_, and PaO_2_/FiO2 ratio. These variables, together with underlying disease, blood gas analysis parameters, time elapsed with cannula, comordibity, Barthel index, and the condition of ventilation, were evaluated in a logistic model as predictors of decannulation.

**Results:**

63 % of patients were successfully decannulated in agreement with our protocol and no one needed to be re-cannulated. Three variables were significantly associated with the decannulation: no pulmonary underlying diseases (OR = 7.12; 95 % CI 1.2–42.2), no mechanical ventilation (OR = 9.55; 95 % CI 2.1–44.2) and period of tracheostomy ≤10 weeks (OR = 6.5; 95 % CI 1.6–27.5).

**Conclusions:**

The positive course of decannulated patients supports the suitability of the weaning protocol we propose here. The strong predictive role of three clinical variables gives premise for new studies testing simpler decannulation protocols.

## Background

Tracheostomy is a common surgical procedure performed to protect airways, to perform bronchial toilet and to wean from Intermittent Positive-Pressure Ventilation (IPPV) in critically ill, ventilator-dependent patients [1–5]. This practice has quickly gained success mostly because of the development of the percutaneous dilational tracheostomy, a technique which can be carried out at the bedside [4]. Indications for tracheostomy generally include a respiratory failure due to a prolonged mechanical ventilation, the need to re-intubate the patient after a failed extubation, and the presence of neurological diseases [3]. No general consensus regarding the timing of tracheostomy has been reached so far, although it is largely accepted that prolonged endotracheal intubation (ETI) should be avoided as much as possible. Furthermore, tracheotomies lasting less than seven days have been associated to a shorter duration of mechanical ventilation and a shorter stay in the intensive care unit (ICU) [6, 7]. In contrast with this evidence, an international multi-center survey carried out in 361 ICU patients revealed that the median length of stay of patients with tracheostomy in the intensive care unit was 21 days, for a total of 36 days of hospitalization. The authors of the survey concluded that tracheostomy is associated with a longer stay, and that the mortality of these patients is similar to that of patients without tracheostomy [3]. On the other hand, the benefits of tracheostomy compared to translaryngeal intubation are remarkable, particularly regarding the better comfort for the patient, the greater ease of bronchial aspiration, the decreasing of resistance, the lower risk of infection, and the easier oral communication and nutrition [8]. Finally, the easier weaning from mechanical ventilation facilitates patient’s transfer from ICU to a non-intensive treatment [9].

To counterbalance the benefits mentioned above, positioning the endotracheal cannula may induce perioperative bleeding, pneumothorax, dislocation, and cannula aspiration. Although complications related to tracheal cannula positioning have been greatly reduced by the latest development of the techniques, late side effects, such as nosocomial infection of tracheal stenosis, and deglutition defects, may occur [10, 11].

In addition, the return to residence of tracheotomised patients is undoubtedly more difficult for caregiver(s), and the survival of these patients has been found to be worse when compared to decannulated patients [12].

Removing tracheostomy is an essential step in rehabilitating patients recovering from critical illness. This achievement is also of considerable importance for the quality of life and for the reduction of the associated costs [13]. Although, at present, no randomised studies have been published on validating criteria for undergoing tracheotomies, a large consensus exists on clinical conditions requiring this procedure, and several reports are available [14–18]. On the contrary, only very few studies have been published on the clinical criteria required for the removal of the endotracheal cannula, many of which based on experts’ opinions [19, 20]. A recent survey carried out on 32 Italian Respiratory Intensive Care Unit (RICU) showed that the proportion of patients decannulated is rather low (22 %), and that there is a notable portion of subjects (26 %) who, even though weaned from mechanical ventilation, were discharged with the tracheotomy tube still applied [21].

Given the evident benefits of tracheostomy in comparison with translaryngeal intubation, and considering the lack of general consensus regarding the timing and conditions for removal, a new decannulation protocol has been developed by the multidisciplinary team of the Unit of Pulmonary Medicine and Rehabilitation of San Raffaele Montecompatri (Rome) and Pulmonary Medicine and Rehabilitation, Villa Delle Querce Hospital, Nemi, (Rome, Italy) on tracheotomised patients admitted to the units [6–9]. Therefore, aim of this study was the evaluation of the efficacy of that protocol and to analyze factors that could predict the success of decannulation.

## Methods

### Study subjects

We retrospectively analyzed 48 medical records of consecutive patients admitted to the Unit of Pulmonary Medicine and Rehabilitation of San Raffaele Montecompatri (Rome) and Pulmonary Medicine and Rehabilitation, Villa Delle Querce Hospital, Nemi, (Rome, Italy) from 2009 to 2014. Twenty-eight patients were admitted from intensive care units (ICU), 14 from other clinical departments, and 6 came directly from home.

The most frequent underlying diseases leading to tracheostomy were: COPD (*n* = 23), ischemic cardiopathy requiring surgical treatment (*n* = 8), respiratory failure following fibrothorax (*n* = 4), and pneumonia (*n* = 2) (Table 1). Subjects affected by neuromuscular diseases were excluded from the study for the technical difficulty of decannulation in these patients. The study was approved by the local Ethical Committee.

**Table 1:** Distribution of underlying diseases cause of tracheostomy

Underlying diseases	Number	Percent
Pulmonary		
COPD	23	47.9
Fibrothorax	4	8.3
Pneumonia	2	4.1
Obstruction Sleep Apnoea Syndrome, Lung cancer, Emopneumothorax, Pulmonary fibrosis	4	8.4
Total	33	68.7
Cardiac		
Cardiac surgery (By-pass)	4	8.3
Heart failure, Cardiac surgery (thoracic-abdominal aneurysm), Cardiac tamponade	3	6.3
Cardiac surgery (valvulopathy)	3	6.3
Total	10	20.9
Abdominal Surgery		
Acute pancreatitis, septic shock, stomach cancer, bowel stroke, peritonitis	4	8.4
Total	4	8.4
Other		
Orthopedic complications	1	2.1
Total	48	100.0

### Study design

The following data were collected at admission: underlying disease, haemogasanalysis parameters (pH, PaO_2_, PaCO_2_, PaO_2_/FiO_2_; Novamedical equipment), time from the tracheostomy (expressed in days), comorbidity (assessed by the Charlson index (CI)), degree of disability (assessed by the Barthel index (BI)), and the presence of mechanical ventilation (yes or not). Ventilated patients were weaned from the ventilator before undergoing to decannulation protocol.

The rehabilitative programme included: active mobilisation of the limbs, electro-stimulation of the quadriceps, abdominal muscle reinforcement, respiratory muscle training, strength retraining via ergometric cycle for the lower limbs and arm ergometer for the upper limbs, bronchial clearing techniques. The protocol for weaning from tracheostomy was designed to choose the best moment of decannulation. The following criteria were considered: clinical stability, expiratory muscle strength assessed by measuring maximal expiratory pressure (MEP) using a portable manometer (Micro Medical Ltd), presence of tracheal stenosis or granulomas via fibrobroncoscopy (nasal passage exam; Olympus BF-P60-fiber Bronchoscope), deglutition function via laryngoscopy and videofluoroscopy (Pentax VNL-1330 ENT Flexible Endoscope), partial pressure of CO_2_ (PaCO_2_), and the ratio between partial pressure of oxygen and the fraction of inspired oxygen (PaO_2_/FiO_2_). If all these requirements were met, a fenestrated cannula was placed and then closed with a cap for a progressively longer time period, up to 48 h. During this process, nocturnal and diurnal oxyhaemoglobin saturation and capnia were monitored (Pulsox 300 i Konica Minolta). In case of efficient cough, absence of significant desaturation, and with PaCO_2_ values < 50 mmHg, the cannula was removed after 72 h; otherwise, a new non-fenestrated cannula was inserted. In Table 2 are specifically reported the criteria for decannulation included in the protocol. At the end of the period of observation patients were divided in two groups: decannulated patients (D) and non-decannulated patients (ND).

**Table 2:** Decannulation protocol

Conditions for decannulation	
All criteria must be satisfied	
Clinical stability	Aemodinamic stability, absence of fever, sepsis or active infection
MEP	>50 cm H_2_O
Nocturnal oxyhaemoglobin desaturation	Absence
PaCO2	<50 mmHg
PaO2/FiO2	>200 (ratio)
Tracheal stenosis and/or granulomas	Absence
Deglutition	Efficient
Patient consent	Positive

*MEP* Maximal Expiratory Pressure

### Analysis

The presence of heterogeneity in the distribution of subjects between the two groups of patients D and ND was tested with the χ square test for all demographic and clinical categorical variables. Continuous variables were compared using the student’s *t*-test for normally distributed variables and the Mann–Whitney *U* test for non-normally distributed variables. In order to identify the decannulation predictors, considering the dichotomic nature of the dependent variable (D or ND), the logistic regression model was applied. The adequacy of the model was tested with the Hosmer-Lemeshow test and by the evaluation of correctly predicted results with the classification analysis.

In regard to covariates included in the analysis, the underlying diseases were grouped into pulmonary and non-pulmonary disease; each hemogasanalysis parameter was categorized in normal or pathological according to the following normal range: pH ≥ 7.36, PaO_2_ between 70 and 80 mmHg, PaCO between 36 and 44 mmHg, PaO_2_/FiO_2_ > 200 mmHg; time to tracheostomy was categorized in ≤10 weeks and >10 weeks (where 10 weeks was the median time after tracheostomy); patients undergoing mechanical ventilation were defined as “ventilated” (V), whereas patients that were autonomous ventilated were defined as “non-ventilated” (NV).

Finally, a cumulative predictor, summing up the number of significant predictive factors was evaluated. This variable was built up according to the result of the logistic regression analysis, i.e. subjects were grouped into four categories: 1) patients without any predictive factor, 2) patients with only one predictive factor, 3) patients with the combination of any two predictive factors, 4) patients with all predictive factors. Statistical significance was set at a value of *P* < 0.050. The SPSS software package version 13.0 (SPSS Inc., Chicago, Illinois, USA) was used for all analyses.

## Results

Demographic and clinical data considered in the study are summarized in Table 3. The study sample was composed by 27 men and 21 women ranging from 38 to 90 years of age (mean = 70.8, sd = 10.4). The level of comorbidity (CI, mean = 4.1, sd = 2.0), and the level of self-sufficiency (BI, mean = 28.1, sd = 27.7) were medium-low. Patients who met the protocol requirement and were therefore decannulated, were 28 (58.3 %).

**Table 3:** Demographic and clinical characteristics of the study group by decannulation category

	Decannulated patients	Non-decannulated patients	Decannulated *vs* non-decannulated patients
	(*N* = 28)	(*N* = 20)	(*P*)
Male, n (%)	17 (60.7)	10 (50.0)	0.46
Age (mean (sd))	68.71 (10.3)	73.65 (10.0)	0.08
Age < = 72 years, n (%)	17 (60.7)	8 (40.0)	0.16
Time elapsed with tracheostomy (days) (mean (sd))	91.61 (110.7)	215.50 (317.9)	0.02*
Time elapsed with tracheostomy < = 10 weeks, n (%)	18 (64.3)	6 (30.0)	0.02*
Admission source			
Home, n (%)	3 (10.7)	3 (15.0)	0.49
Ward, n (%)	10 (35.7)	4 (20.0)	
ICU, n (%)	15 (53.6)	13 (65.0)	
Length of stay (mean (sd))	46.79 (33.6)	47.20 (19.1)	0.39
Pulmonary disease, n (%)	15 (53.6)	18 (90.0)	0.01*
Charlson Index (mean (sd))	3.68 (2.2)	4.60 (191.0)	0.05
Mechanical Ventilation at admission, n (%)	10 (35.7)	17 (85.0)	<0.01*
Weaning from Mechanical Ventilation (*n* = 27), n (%)	10 (35.7)	4 (20.0)	
Barthel Index (mean (sd))	31.96 (31.2)	17.85 (19.9)	0.15
pH (mean (sd))	7.43 (0.1)	7.40 (0.0)	0.04*
PaCO2 (mmHg) (mean (sd))	46.07 (11.0)	49.30 (15.2)	0.44
PaO_2_ (mmHg) (mean (sd))	64.03 (10.7)	76.10 (18.9)	0.02*
PaO2/FiO_2_ (mean (sd))	255.46 (60.9)	270.00 (97.0)	0.50

*Significant difference

According to the χ square test, gender and admission source were not associated to decannulation. Similarly, neither Barthel index, nor haemogasanalysis parameters according to the Mann–Whitney test resulted to be significantly different in the two groups of patients (Table 3). A signal for a diversity of the two groups was found with regards to the age (*p* = 0.080) and the Charlson index at hospitalization (CI, *p* = 0.054) (Table 3).

A significant difference was found between patient group affected by pulmonary diseases and patient group affected by non-pulmonary diseases (*p* = 0.007). In fact, only 45 % of patients affected by pulmonary diseases achieved the final decannulation at the end of rehabilitation programme, against 86 % of patients affected by non-pulmonary diseases. Further significant associations with decannulation were found with patient characteristics, i.e., (I) the ventilation parameter (almost 86 % of non-ventilated patients were decannulated, against 37 % of ventilated patients, *p* = 0.001); (II) the median pH value, which was significantly higher in D (pH 7.43) compared to ND (pH 7.40) (*p* = 0.038); (III) the PaO_2_, which, on the contrary, was significantly lower in D (PaO_2_ 45.6) than in ND (PaO2 47.1) (*p* = 0.018), and (IV) the period spent with the cannula before the rehabilitation, which was significantly shorter in D (2 months) compared to ND (3 months and 23 days) (*p* = 0.016) (Table 3).

The results of the logistic regression analysis, showed as only the ventilation, the underlying diseases, and the duration of the tracheostomy were associated to the decannulation after adjusting for confounding. In particular, non-ventilated patients were almost 10 times more frequently decannulated than mechanically ventilated patients (OR = 9.55; CI95 % = 2.07–44.18), patients affected by a non-pulmonary diseases were 7 times more frequently decannulated than patients with pulmonary diseases (OR = 7.12; CI95 % = 1.20–42.17), and finally, patients with tracheostomy for less than 10 weeks were 6 times more frequently decannulated than patients which were incannulated for a longer period (OR = 6.52; CI95 % = 1.55–27.49). No other variables were found to be significantly associated with the outcome. No significant interaction was found between the predictive variables. Given the excess of empty cells, the saturated model failed to reach an adequate fitting. The results reported in Table 4, therefore, summarize estimates from two separated models, one including mechanical ventilation and pulmonary disease, the other mechanical ventilation and duration of the tracheostomy. The Hosmer and Lemoshow goodness of fit test revealed a good calibration of both models (model 1: *χ*2 = 0.545, df = 2, *p* = 0.762; model 2: *χ*2 = 0.401, df = 2, *p* = 0.818). Moreover, model 1 correctly classifies 77.1 % of subjects, model 2 75.0 %.

**Table 4:** Clinical variables predicting decannulation (Logistic regression model)

Variables		Number	Odds ratio	CI_95%_ for OR		*P*
		(Decannulated/Non decannulated)		Lower	Upper	
Mechanical Ventilation	Absence	18/3	1.00	-	-	-
	Presence	10/17	9.55	2.07-	44.18	<0.01*
Pulmonary diseases	Absence	13/2	1.00	-	-	-
	Presence	15/18	7.12	1.20-	42.17	0.03*
Time elapsed with tracheostomy	<= 10 weeks	18/6	1.00	-	-	-
	>10 weeks	10/14	6.52	1.55-	27.49	0.01*

*Significant difference

**Fig. 1:**
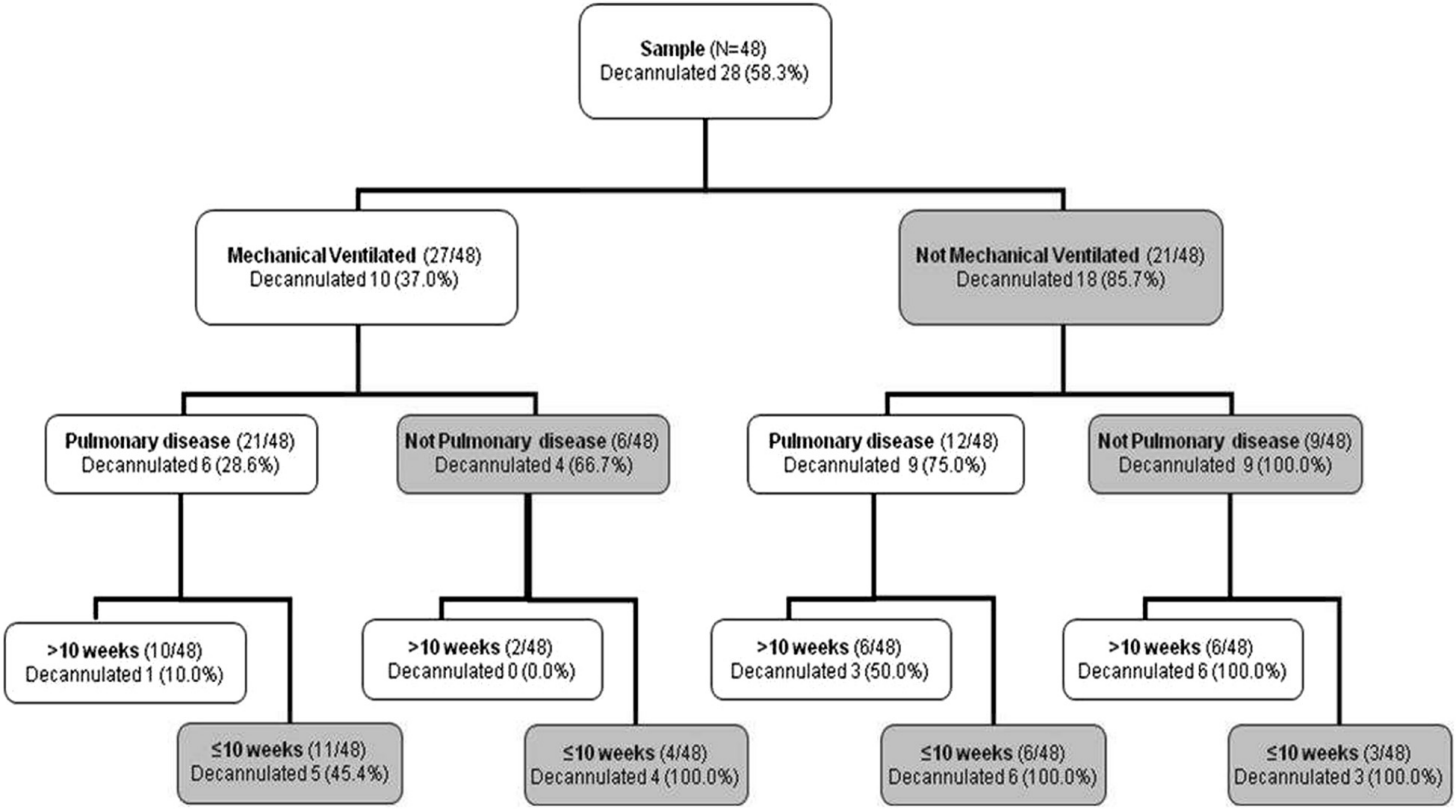
Flow-chart of patients weaned from tracheostomy by presence of mechanical ventilation, underlying disease, and the timing of tracheostomy

In order to understand how significant predictors impacted on decannulation, the distribution of decannulated patients was evaluated according to these variables. The rehabilitation programme was successfully concluded by the 58 % of patients. Among these, 86 % was able to breath autonomously. Focusing on ventilation, 86 % of NV patients were weaned from tracheostomy against 37 % of V patients. The concomitant presence of pulmonary disease had a pivotal role in the success of weaning from tracheostomy (pulmonary disease patients: V 29 %, NV 75 %; non-pulmonary disease patients: V 67 %, NV 100 %). In addition, when patients were evaluated according to the time with the cannula, independently from other features, a higher percentage of decannulation was registered in patients with a history of tracheostomy lower than 10 weeks compared to the groups of patients that maintained cannula for more than 10 weeks. As a direct consequence of these figures, the success of the rehabilitative programme was less frequent in the group of patients needing mechanical ventilation, affected by pulmonary disease and with cannula for more than 10 weeks. On the other hand, a higher percentage of weaning from tracheostomy was registered in the NV patients and in subjects not affected by pulmonary diseases, indipendently of time with cannula. In fact, all patients without pulmonary disease who kept the cannula for less than 10 weeks were successfully decannulated (Fig. 1).

Considering only predictive factors of decannulation (non pulmonary underlying disease, absence of mechanical ventilation, and time with cannula lower than 10 weeks), patients with no predictive factors were 10 and only one of them was decannulated (10 %); patients with two predictive factors, were 19 and among these 8 (42 %) were decannulated; finally patients with all three predictive factors were 19, and all of them were decannulated (100 %).

The total number of mechanically ventilated patients at the end of the rehabilitation programme was 27 (56 %), 14 of them (51 %) were weaned from V, and 10 (71 % of weaned from V) were also weaned from tracheostomy. Finally, as expectd, any patients not weaned from V were weaned from tracheostomy.

## Discussion

The results of this study suggests that weaning from tracheostomy is associated with few variables, the underlying diseases, the presence of mechanical ventilation, and the period of time with the cannula, that can be easily evaluated in patients undergoing a rehabilitation programme. The other major finding was the reliability of the specific decannulation protocol, developed in our unit, which may help in optimizing weaning from tracheostomy in critical care patients.

The findings of this work revealed as the clinical features that should be considered to undergo decannulation can be minimized, and all of them are objective and easily proven in patients. Nevertheless, other few variables such as the age of patients, the CI, the pH, and the PaO_2_ showed a weak association with decannulation, and considering the sample size and the retrospective nature of the study, it is not possible to exclude that these non-significant variables could play a role in the decision to wean from tracheostomy.

At present, in contrast with the general consensus concerning the timing for performing tracheostomy, studies on the correct moment for a definitive decannulating patients with tracheostomy are few and mainly based on the personal experience of individual authors. Godwin and Heffner firstly evaluated the ability to speak and to eat, and then suggested decannulation after positive results with closed cannula [22]. Instead, Ceriana and collaborators proposed a more organic approach in a group of 108 tracheotomised patients with prolonged mechanical ventilation; using a simple decision flow-chart they were able to remove cannula in almost 80 % of subjects [23]. More recently, Newman and collaborators evaluated tracheostomy removal in a small group of patients recovered in a palliative care unit [24]. In addition, a recent international survey involving 309 doctors and respiratory therapists highlighted as the most popular criteria for deciding decannulation was the patient level of consciousness, cough effectiveness, secretions, and oxygenation [13]. In a multicentre, perspective and observational study carried out in the United States with patients subjected to prolonged mechanical ventilation, 54 % of this group was weaned from ventilatory support and only 59 % of them was able to subsequently be decannulated. However, this study did not mention the procedures adopted for establishing decannulation feasibility [25, 26]. More recently, O’Connor showed that the lack of a proper swallowing evaluation and an earlier transfer from acute care facilities predicted inability to decannulate [27].

In addition to the lack of studies concerning the correct moment for resolving tracheostomy and decannulation, little else exists in the literature regarding factors which can predict the decannulation success. Christopher and coll. included the ability to produce a vigorous cough and the absence of aspiration in the decannulation predictors [28], while Bach and colleagues demonstrated that only peak cough flow represented a good prediction index for decannulation, although the study was made exclusively in neuromuscular tracheostomized patients [19, 28]. O’Connor noted that those patients who could not be decannulated had been more precociously tracheotomised and had shorter stays in the intensive care unit [27].

Removing cannula represents a decisive step in the rehabilitative process of critical patients, but despite the consensus on this priority few studies have addressed this issue, the majority of which were based on subjective and not standardised criteria.

Modern medicine is based on scientific evidences and, therefore, even in the absence of established guidelines, the definition of institutional protocols represents a step forward with respect to simple clinical assessment.

The decannulation protocol described in this paper reflects our clinical experience in pulmonary medicine and rehabilitation, therefore a valuable knowledge as confirmed by scientific literature which shows as doctors working in critical care units, in rehabilitation units, or in weaning centres had a higher percentage of decannulated patients compared to those in long term care [13, 29].

Our protocol was based on the objective evaluation of characteristics such as expiratory muscle strength and cough efficacy, absence of dysphagia, absence of granulomas and stenosis, good oxygenation and normocapnia. In order to assess the capacity of removing secretions, we used the MEP test instead of the peak cough expiratory flow (PCEF) as the latter is a better predictor of cough efficacy selectively in neuromuscular patients, which were deliberately excluded from the study [19].

Importance was also attributed to the evaluation of dysphagia for which we utilised the videofluoroscopy adding the “blue dye test”, which is still the most cited test in the literature [30–32]. The utilisation of a fenestrated cannula seems to be a better approach towards subsequent decannulation in comparison to the use of lower calibre cannulas or of mini-trachs, as it permits greater patient comfort allowing phonation and furthermore providing the possibility to progressively restore physiological airway potency [23]. In addition, we considered comorbidity and took note of BI, believing that associated diseases and the level of disability could, in some way, influence the good outcome of the procedure.

Removing the cannula is undoubtedly a critical procedure for its positive effects on the quality of life, on survival, as well as on the home management. The absence of standardised protocols may result in an underutilisation of this option, most likely denying decannulation even to those subjects who could otherwise be comfortably decannulated. According to our experience good clinical stability (mostly determined by capnia levels), sufficient respiratory musculature performance and therefore cough efficacy, normal airway patency and absence of dysphagia, ease the process of removing cannula in the majority of cases. Furthermore, the level of functional disability and underlying diseases seem to influence the good outcome of the procedure. This assessment, although to be confirmed in extensive case study, is based on the clinical evidence that the early application of active and passive mobilisation in highly critical patients, even those with tracheostomy, has a positive impact on the outcome [32–34]. The suitability of our findings is certainly limited by the pilot and retrospective characteristics of the study; the next phase will be to further validate the decannulation protocol and to conform the predictive variable of weaning from tracheostomy to a prospective study design, based on a larger sample of patients undergoing pulmonary rehabilitation.

## Conclusions

In conclusion, using a specific decannulation protocol we identified in a group of patients undergoing a pulmonary rehabilitation programme a minimal set of predictive variables, i.e. underlying disease, mechanical ventilation, and time with cannula, that can be efficiently used to address the clinical procedures of weaning from tracheostomy. Although the protocol proved its suitability in this specific subset of patients, larger prospective studies are needed for further validation.
